# Learning Curves for Robotic-Assisted Ventral Hernia Repair

**DOI:** 10.1001/jamanetworkopen.2024.48521

**Published:** 2024-12-03

**Authors:** Wei San Loh, Ryan A. Howard, Brian T. Fry, Jyothi R. Thumma, Edward C. Norton, Justin B. Dimick, Kyle H. Sheetz

**Affiliations:** 1 Center for Healthcare Outcomes and Policy, University of Michigan, Ann Arbor; 2 Department of Surgery, University of Michigan, Ann Arbor; 3 Department of Health Management and Policy, University of Michigan, Ann Arbor; 4 Department of Economics, University of Michigan, Ann Arbor; 5 Surgical Innovation Editor, *JAMA Surgery*, Chicago, Illinois

## Abstract

**Importance:**

Although the use of robotic-assisted ventral hernia repairs has increased significantly over the last decade, the experience surgeons need to achieve comparable outcomes with more established laparoscopic and open approaches has not been well characterized.

**Objective:**

To estimate the learning curves for robotic-assisted ventral (incisional and umbilical) hernia repair.

**Design, Setting, and Participants:**

This retrospective cohort study included Medicare fee-for-service patients (≥18 years) enrolled in Medicare Part A and Part B with no managed care undergoing ventral hernia repairs between 2010 and 2020. Multivariate logistic regression was used to estimate reoperation rates for hernia recurrence, adjusting for patient demographics, comorbidities, mesh placement, and hospital characteristics. Analyses were conducted from October 2023 to July 2024.

**Exposure:**

Incremental robotic-assisted hernia repair volume.

**Main Outcome and Measure:**

Reoperation for hernia recurrence within 7 years of index hernia repair.

**Results:**

This study comprised 160 379 Medicare patients (mean [SD] age, 69 [11] years), of whom 93 272 (58.2%) were female, 13 799 (8.6%) were Black, 3124 (2.0%) were Hispanic, and 138 311 (86.2%) were White. Among these patients, 12 609 (7.9%) underwent robotic-assisted hernia repairs, 32 337 (20.2%) laparoscopic repairs, and 115 433 (71.9%) open repairs. This study also included 23 580 surgeons, with 5074 performing robotic-assisted hernia repairs. The national reoperation rates for hernia recurrence after laparoscopic and open repairs were 12.5% (95% CI, 12.06%-12.94%) and 12.9% (95% CI, 12.70%-13.15%), respectively. Reoperation rates decreased as the number of robotic-assisted cases increased, from 14.2% (95% CI, 13.34%-14.97%) at 10 cases to 7.6% (95% CI, 6.75-8.37) at 50 cases. Surgeons needed at least 19 (95% CI, 16-22) robotic-assisted hernia repairs or 16 (95% CI, 13-19) repairs to achieve outcomes equivalent to those of laparoscopic and open procedures. Few surgeons (285 surgeons [5.7%]) performed enough robotic-assisted ventral hernia repairs to achieve necessary volume nationally to overcome the learning curve.

**Conclusions and Relevance:**

In this cohort study of 23 580 surgeons who performed ventral hernia repairs between 2010 and 2020, increasing experience with robotic-assisted hernia repairs was associated with improved long-term reoperation rates for hernia recurrence. However, the majority of surgeons did not perform enough cases to reach reoperation rates equivalent to more established laparoscopic and open approaches.

## Key Points

**Question:**

How many surgical cases are associated with achieving comparable long-term outcomes after robotic-assisted ventral hernia repair compared with laparoscopic and open approaches?

**Findings:**

In this cohort study of 23 580 surgeons, approximately 19 robotic-assisted hernia repairs were associated with comparable long-term reoperation rates for hernia recurrence as laparoscopic and open repairs; however, fewer than 6% of surgeons exceeded that volume.

**Meaning:**

These results suggest that while increasing experience with robotic-assisted hernia repairs is associated with improved long-term outcomes, most surgeons do not perform enough cases to reach equivalent reoperation rates for hernia recurrence to more established laparoscopic and open approaches.

## Teaser

This cohort study evaluates the learning curves for robotic-assisted ventral hernia repair using data from Medicare fee-for-service patients from 2010 to 2020.

## Introduction

The use of robotic-assisted ventral hernia repair has increased more than any other surgical procedure over the last decade.^1^ For instance, among Medicare beneficiaries, the proportion of robotic-assisted ventral hernia repairs increased from 0.5% to 13.8% between 2010 and 2018, while more commonly used approaches such as laparoscopic and open approaches decreased from 25.8% to 15.9% and 73.7% to 70.2%, respectively.^2^ Hospital credentialing bodies often require surgeons to accumulate experience with the robotic platform before obtaining privileges to perform hernia repairs (or any other surgery) using the approach.

Despite the rapid adoption of robotic-assisted ventral hernia repair, it remains unclear how much experience is needed by surgeons who perform robotic-assisted surgery to achieve equivalent outcomes compared with more established laparoscopic and open approaches. First, existing studies comparing robotic-assisted, laparoscopic, and open ventral hernia repairs focused only on the recurrence rates across these procedures but not the surgeons’ learning curve for robotic-assisted surgery.^3,4^ Second, existing randomized clinical trials comparing patients’ short-term recurrence rates across robotic-assisted and laparoscopic approaches were conducted with sample populations, using only surgeons with extensive experience in robotic-assisted and laparoscopic ventral hernia repair and hence are not easily generalizable to all surgeons.^5,6^ Third, existing studies have not examined the experience necessary for robotic-assisted hernia repairs to reach equivalent (or better) outcomes than more established approaches like laparoscopic and open repairs.

We aim to characterize the learning curve for robotic ventral hernia repairs with respect to long-term reoperation for hernia recurrence. Using national Medicare data, our estimates reflect a representative cohort of surgeons with accurate long-term patient follow-up, given the near universal continuous enrollment and high retention level among beneficiaries. Because the use of robotic-assisted surgery began to increase rapidly after 2010, we captured individual surgeons as they accumulated experience with the robotic-assisted approach from 2010 to 2020. Our findings will therefore fill the literature gap in surgeons’ learning curve for robotic-assisted ventral hernia repairs.

## Methods

### Data Source and Study Population

This study was deemed exempt from review and informed consent by the University of Michigan institutional review board due to the use of retrospective deidentified data. We followed the Strengthening the Reporting of Observational Studies in Epidemiology (STROBE) reporting guideline for cohort studies.

We used patient-level data from 100% Medicare fee-for-service patients who are enrolled in Medicare Part A and Part B with no managed care. Detailed cohort selection methods can be found in the study by Fry et al,^3^ which used a similar process. Among these patients, we included Medicare beneficiaries aged 18 years or older who underwent elective inpatient ventral hernia repairs (ie, incisional or umbilical) between January 1, 2010, and December 31, 2020.

Individuals were identified by matching *Current Procedural Terminology* (*CPT*) codes and *International Classification of Diseases Ninth and Tenth Edition* (*ICD-9* and* ICD-10*) procedure codes. These procedure codes were then cross-referenced with relevant *ICD-9* and *ICD-10* diagnosis codes associated with ventral (incisional or umbilical) hernias (see eTable 1 in Supplement 1). The transition from *ICD-9* to *ICD-10* occurred in October 2015, but the coding changes related to ventral hernia repairs were minimal.

We linked inpatient index hernia surgical procedures with part B line-level claims to identify the National Provider Identification of performing surgeons. For each surgeon, we identified their earliest claim during the study period as their index hernia operation, arranged chronologically to assess reoperation rates for hernia recurrence based on the surgeon’s operation volume.

The surgeon’s patient volume was inflated using the proportion of Medicare patients at the hospital, better reflecting the total number of patients per surgeon, not limited to Medicare patients.^7^ We estimated hospital total case volume by extrapolating the Medicare hernia volume using the hospital level Medicare percentages for each year from 2010 to 2020. We calculated the percentage of Medicare patients out of total hospital admissions for each hospital using the ratio of hospital total Medicare discharges to the total admissions from American Hospital Association Annual Survey data.

### Outcome Measures and Explanatory Variables

The primary outcome was the rate of reoperation for hernia recurrence within 7 years following the index hernia repair. The primary exposure was incremental surgeon case volume. To ensure that the results were robust, the analysis included covariates such as patient’s age, sex, self-reported race and ethnicity (American Indian or Alaska Native, Asian or Pacific Islander, Black, Hispanic, non-Hispanic White, Other race, and unknown), and Elixhauser comorbidities. Race and ethnicity were included as a social construct to account for disparities. Other covariates included hernia repair approach (ie, robotic, laparoscopic, or open), use of mesh or myofascial flap, and hernia subtype (umbilical or incisional).

### Statistical Analysis

A multivariate logistic regression model was used to estimate the association between surgeon experience and the learning curve for robotic-assisted ventral hernia repair, with reoperation for hernia recurrence within 7 years as the primary outcome and surgeons’ operation volume as the primary exposure. Marginal effects were calculated to determine the probability of reoperation for hernia recurrence within 7 years based on surgeons’ robotic-assisted operation volume. The same model was used to compute the average reoperation rates for patients undergoing either laparoscopic or open approach.

Linear splines were incorporated into the regression model with knots placed at 20, 30, and 40 cases to divide the regression curve into meaningful segments and account for potential nonlinearity in the association between surgeons’ volume and reoperation risk. To account for the shorter follow-up period (less than 7 years) for patients from 2017 to 2021, we conducted a sensitivity analysis using 4-year reoperation rates for hernia recurrence (see eMethods and eFigure in Supplement 1).

All statistical tests were 2-sided, and a *P* value less than .05 was considered statistically significant. Analyses were conducted using Stata version MP18 (StataCorp) from October 2023 to July 2024.

## Results

### Patient and Surgeon Characteristics

**Figure 1.**
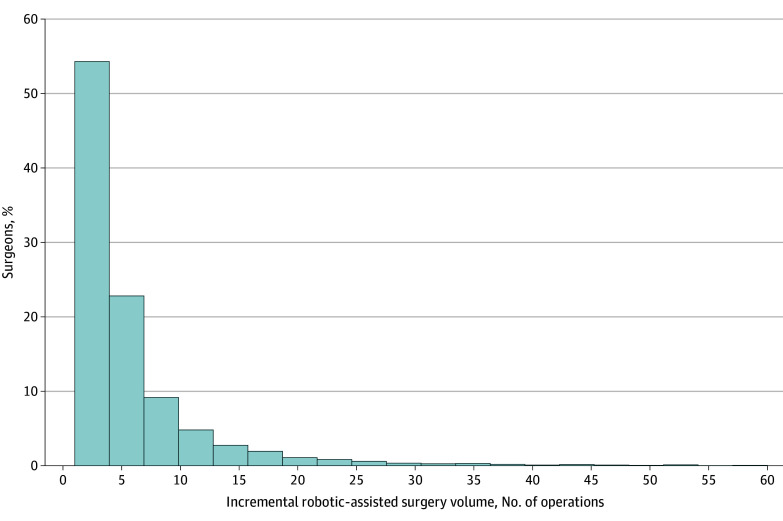
Distribution of Surgeons' Robotic-Assisted Case Volume (2010-2020) Histogram of the proportion of surgeons performing robotic-assisted hernia repairs grouped by total volume from 2010 to 2020. The x-axis reflects total volume derived from the ratio of hospital total Medicare discharges to total admissions from American Hospital Association data.

This study comprised 160 379 Medicare patients (mean [SD] age, 69 [10.8] years), of whom 93 272 (58.2%) were female, 13 799 (8.6%) were Black, 3124 (2.0%) were Hispanic, and 138 311 (86.2%) were White (Table 1). Among these patients, 12 609 (7.9%) underwent robotic-assisted hernia repairs, 32 337 (20.2%) underwent laparoscopic repairs, and 115 433 (71.9%) underwent open repairs. This study also included 23 580 surgeons, with 5074 performing robotic-assisted hernia repairs. The proportion of robotic-assisted hernia repairs increased from 2.1% to 21.9% between 2010 and 2020, whereas laparoscopic and open approaches decreased from 23.8% to 11.9% and 74.2% to 66.2%, respectively (see eTable 2 in Supplement 1). During the study period, approximately 4450 surgeons (88%) performing robotic procedures conducted 10 or fewer robotic-assisted hernia repairs (Figure 1).

**Table 1.** Patient Characteristics by Operative Approach

Characteristic	Patients, No. (%)				*P* value^a^
	All (N = 160 379)	Robotic (n = 12 609)	Laparoscopic (n = 32 337)	Open (n = 115 433)	
Age, mean (SD), y	69 (10.8)	69.9 (8.9)	68.5 (11.1)	69.0 (10.9)	.001
Sex					
Male	67 107 (41.8)	6831 (54.2)	12 448 (38.5)	47 828 (41.4)	.001
Female	93 272 (58.2)	5778 (45.8)	19 889 (61.5)	67 605 (58.6)	
Race and ethnicity					
American Indian or Alaska Native	975 (0.6)	59 (0.5)	202 (0.6)	714 (0.6)	.11
Asian or Pacific Islander	867 (0.5)	88 (0.7)	152 (0.5)	627 (0.5)	.01
Black	13 799 (8.6)	1046 (8.3)	2689 (8.3)	10 064 (8.7)	.03
Hispanic	3124 (2.0)	225 (1.8)	588 (1.8)	2311 (2.0)	.04
Non-Hispanic White	138 311 (86.2)	10 835 (85.9)	28 108 (86.9)	99 368 (86.1)	.001
Other^b^	1627 (1.0)	142 (1.1)	325 (1.0)	1160 (1.0)	.43
Unknown	1676 (1.1)	214 (1.7)	273 (0.8)	1189 (1.0)	.001
Comorbidities					
Congestive heart failure	12 573 (7.8)	917 (7.3)	2357 (7.3)	9299 (8.1)	.001
Valvular disease	7440 (4.6)	555 (4.4)	1508 (4.7)	5377 (4.7)	.40
Peripheral vascular disease	8260 (5.2)	508 (4.0)	1568 (4.8)	6184 (5.4)	.001
Other neurological disorders	7719 (4.8)	492 (3.9)	1507 (4.7)	5720 (5.0)	.001
Chronic pulmonary disease	37 621 (23.5)	2668 (21.2)	7976 (24.7)	26 977 (23.4)	.001
Diabetes without chronic complications	36 418 (22.7)	2403 (19.1)	8025 (24.8)	25 990 (22.5)	.001
Diabetes with chronic complications	9733 (6.1)	1071 (8.5)	1688 (5.2)	6974 (6.0)	.001
Hypothyroidism	24 835 (15.5)	1854 (14.7)	5180 (16.0)	17 801 (15.4)	.001
Kidney failure	18 597 (11.6)	1398 (11.1)	3286 (10.2)	13 913 (12.1)	.001
Liver disease	7125 (4.4)	424 (3.4)	1552 (4.8)	5149 (4.5)	.001
Rheumatoid arthritis/collagen vascular disease	5072 (3.2)	352 (2.8)	1014 (3.1)	3706 (3.2)	.03
Obesity	37 698 (23.5)	3270 (25.9)	7564 (23.4)	26 864 (23.3)	.001
Weight loss	5113 (3.2)	262 (2.1)	528 (1.6)	4323 (3.7)	.001
Fluid and electrolyte disorders	24 095 (15.0)	1557 (12.3)	3536 (10.9)	19 002 (16.5)	.001
Deficiency anemias	18 227 (11.4)	1204 (9.5)	2904 (9.0)	14 119 (12.2)	.001
Depression	19 399 (12.1)	1395 (11.1)	4240 (13.1)	13 764 (11.9)	.001
Hypertension	107 540 (67.1)	8585 (68.1)	21 931 (67.8)	77 024 (66.7)	.001
Hernia type					
Incisional	130 210 (81.2)	7627 (60.5)	28 254 (87.4)	94 329 (81.7)	.001
Umbilical	30 169 (18.8)	4982(39.5)	4083 (12.6)	21 104 (18.3)	.001

^a^ *P* values shown here are for analysis of variance or Pearson χ^2^ test depending on the variable type. The *P* values test the equality of percentages or means across robotic, laparoscopic, and open approaches.

^b^ Other race includes racial and ethnic groups other than American Indian or Alaska Native, Asian or Pacific Islander, Black, Hispanic, and non-Hispanic White.

### Robotic-Assisted Hernia Repair Learning Curves and Comparison With Laparoscopic and Open Benchmarks

**Figure 2.**
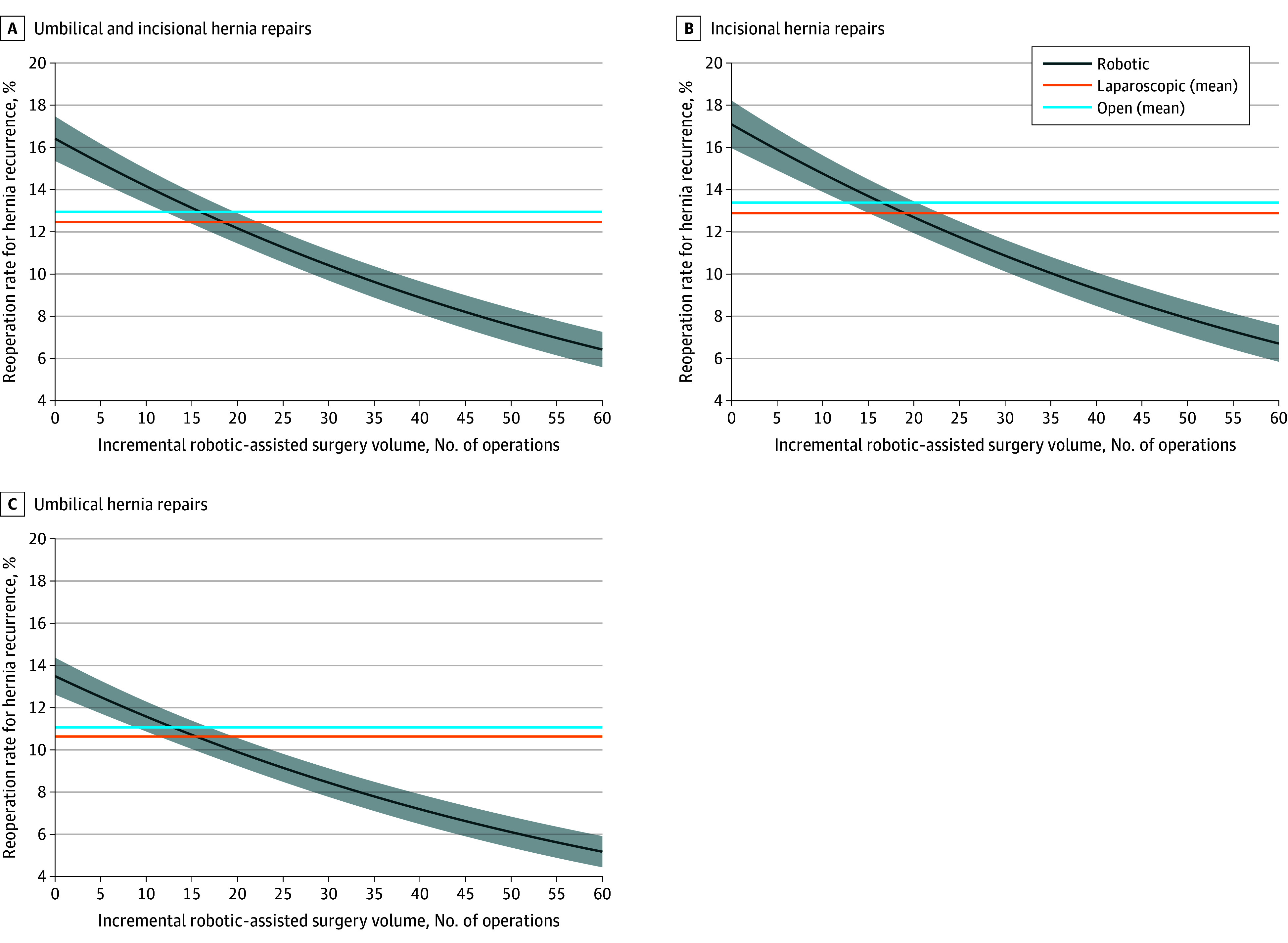
Learning Curves for Different Types of Robotic-Assisted Hernia Repair The learning curves for robotic-assisted hernia repairs (incisional, umbilical, or both incisional and umbilical) show adjusted reoperation rates for hernia recurrence by surgeons’ incremental experience with the approach. The 2 horizontal lines represent the national average reoperation rates for hernia recurrence using laparoscopic or open approach. Data are derived from Medicare fee-for-service patients enrolled in Medicare Part A and Part B (excluding managed care) from 2010 to 2020. The x-axis reflects total volume derived from the ratio of hospital total Medicare discharges to total admissions from American Hospital Association data.

Patient reoperation rates for ventral hernia repairs (umbilical and incisional) using the robotic-assisted approach improved with surgeons’ increasing experience. The reoperation rate for each incremental number of robotic repairs was compared with the average reoperation rates for laparoscopic (mean, 12.5%; 95% CI, 12.06%-12.94%) and open (mean, 12.9%; 95% CI, 12.70%-13.15%) procedures (see Figure 2). Reoperation rates for ventral hernia repairs (umbilical and incisional) using the robotic-assisted approach were lower as the number of cases increased, with reductions of 2.0% (95% CI, 1.89-2.09) after 20 cases, 3.8% (95% CI, 3.65-3.84) after 30 cases, 5.3% (95% CI, 5.22-5.32) after 40 cases, and 6.6% (95% CI, 6.59-6.60) after 50 cases, compared with the baseline reoperation rate of 14.2% at 10 cases (Table 2). Reoperation rates of robotic-assisted hernia repair became equivalent to those who underwent laparoscopic ventral hernia after 19 (95% CI, 16-22) cases. Similarly, it took surgeons 16 (95% CI, 13-19) robotic-assisted cases to achieve equivalent reoperation rates for hernia recurrence for those who underwent open repair. When comparing robotic-assisted with laparoscopic and open approaches, 131 (2.6%) and 285 (5.7%) surgeons had performed high enough robotic-assisted volume to have lower reoperation rates for hernia recurrence than those using the laparoscopic and open approaches, respectively (Table 3).

**Table 2.** Reoperation Rates for Robotic-Assisted Hernia Repairs Based on Surgeons’ Cumulative Experience

Repair type and cumulative volume of robotic-assisted repairs, every 10 cases	Reoperation rate within 7 y of index surgery, % (95% CI)	Change in reoperation rate relative to baseline, % (95% CI)^a^
All repairs		
10	14.2 (13.3-15.0)	1 [Reference]
20	12.2 (11.5-12.9)	2.0 (1.9-2.1)
30	10.4 (9.7-11.1)	3.8 (3.7-3.8)
40	8.9 (8.1-9.7)	5.3 (5.2-5.3)
50	7.6 (6.8-8.4)	6.6 (6.59-6.60)
Umbilical hernia repairs		
10	11.6 (10.9-12.3)	1 [Reference]
20	9.9 (9.2-10.6)	1.7 (1.6-1.7)
30	8.4 (7.8-9.1)	3.2 (3.1-3.2)
40	7.2 (6.5-7.9)	4.4 (4.39-4.40)
50	6.1 (5.4-6.8)	5.5 (5.45-5.50)
Incisional hernia repairs		
10	14.8 (13.9-15.6)	1 [Reference]
20	12.7 (11.9-13.4)	2.1 (2.0-2.2)
30	10.9 (10.1-11.6)	3.9 (3.8-4.0)
40	9.3 (8.5-10.1)	5.5 (5.4-5.6)
50	7.9 (7.1-8.7)	6.9 (6.8-6.9)

^a^ The first 10 cases are the baseline.

**Table 3.** Learning Curves for Robotic-Assisted Hernia Repairs Compared With Laparoscopic or Open Benchmarks

Hernia type	Laparoscopic or open benchmarks		Surgeons better than benchmark using robotic assistance, No. (%)
	Surgery volume (middle 50%)	Reoperation rate within 7 y of index surgery, % (95% CI)	
All hernia repairs	Laparoscopic	11.1 (10.49-11.61)	131 (2.6)
	Open	12.8 (12.42-13.17)	285 (5.7)
Umbilical hernia repairs	Laparoscopic	9.3 (8.73-9.87)	151 (3.0)
	Open	10.8 (10.36-11.27)	373 (7.4)
Incisional hernia repairs	Laparoscopic	11.5 (10.87-12.04)	117 (2.3)
	Open	13.3 (12.86-13.65)	285 (5.7)

Similar outcomes were observed when stratifying by ventral or incisional and umbilical hernia repairs separately (Figure 2). Surgeons needed to perform 13 (95% CI, 11-16) cases of robotic-assisted umbilical hernia repair to achieve equivalent reoperation rates for hernia recurrence as those who underwent repair via an open approach. The reoperation rates of robotic-assisted umbilical hernia repair achieved equivalence to those who underwent laparoscopic umbilical hernia repairs after 16 (95% CI, 12-47) cases. For incisional hernia repairs, it took surgeons 20 (95% CI, 17-22) cases or 17 (95% CI, 16-18) cases to reach equivalent reoperation rates for hernia recurrence as patients who underwent laparoscopic or open incisional hernia repairs, respectively.

## Discussion

This study used national Medicare data to characterize the learning curve associated with robotic-assisted ventral hernia repair. We found that reoperation rates for hernia recurrence decreased as surgeons achieved more robotic-assisted experience. However, few surgeons nationally were performing enough robotic-assisted ventral hernia repairs to achieve equivalent average reoperation rates for hernia recurrence to either laparoscopic or open approach. These data suggest that more deliberate training and credentialing are needed to ensure more surgeons have adequate experience to overcome the learning curve.

Our results are consistent with previous studies on the learning curve of robotic-assisted approaches for both groin and ventral hernia repairs, which demonstrate a range of 25 to 43 cases to achieve proficiency.^8,9,10,11,12^ However, these studies base proficiency largely through operative time, which does not take into account patient complexity, varying operative techniques, and perhaps most importantly, postoperative outcomes. Given that most patients seek hernia repair to improve their quality of life or relieve bothersome symptoms, reoperation for hernia recurrence is likely the most important outcome for patients and perhaps the best indicator of the quality of hernia repair. Our study builds on prior work^3^ and is the first we know of to evaluate learning curves for the robotic platform in relation to rates of operative ventral hernia recurrence, and more specifically, in relation to the hernia recurrence reoperation rates for established laparoscopic and open approaches.

We found that most surgeons did not reach our calculated case number thresholds to overcome the learning curve associated with robotic-assisted ventral hernia repair. Nearly 90% of surgeons had fewer than 10 total robotic-assisted repairs from 2010 to 2020, even after inflating volumes to adjust for hernia repairs performed on non-Medicare patients. Recent population-level data demonstrate that despite increasing use of the robotic platform, only 30% of all inpatient and outpatient ventral hernia repairs were performed in a minimally invasive fashion in 2019.^13^ While our study does not include outpatient ventral hernia repairs, it highlights the need for more intentional exposure and training pathways in order for resident trainees and established surgeons to overcome learning curve thresholds for robotic-assisted ventral hernia repair.

Furthermore, even with dedicated robotic curriculum, it remains unclear whether general surgery trainees will be able to obtain adequate case numbers without further advanced hernia fellowship training.^14,15^ Minimally invasive surgery is great for hernia, but it is currently underutilized. In experienced hands, robotic-assisted repairs may offer patients greater access to minimally invasive surgery or more complex repairs than have been traditionally offered laparoscopically. However, for surgeons looking to gain experience with robotic-assisted surgery, even routine operations like umbilical hernia repair come with new learning curves that should be discussed with patients and acknowledged by hospital credentialling bodies.

This study has important implications for patients, surgeons, and policymakers. For patients undergoing robotic-assisted ventral hernia repair, our results suggest that seeking out surgeons who perform a high volume of robotic cases may reduce the likelihood of future reoperation for hernia recurrence. For surgeons, our results provide procedure-specific benchmark case numbers to help guide the path toward proficiency. However, increasing robotic-assisted hernia repair volumes may come at the expense of obtaining sufficient case numbers for laparoscopic and open approaches, and thus trainees should strive to obtain a diverse portfolio of translatable skills. Finally, for policymakers, these results may inform creation of operative approach-specific case number minimums for surgical residency graduation requirements. For more senior surgeons, they may guide more deliberate adoption of the robot into independent practice after training. Currently, general surgery residents are required to graduate with 85 combined hernia cases (ventral, groin, and so forth) across 5 years of clinical training; however, there are no requirements for case numbers by operative approach.^16^ For independent surgeons integrating the robot into their existing practices, this study’s learning curve thresholds far exceed the standard requirement for proctored robotic-assisted cases before initial credentialing and privileging.^17^ Refining general surgery residents’ training to incorporate operative approaches for specific disease processes would likely help more surgeons reach proficient case numbers and improve patient outcomes.

### Limitations

Several limitations should be considered in interpreting our findings. First, the open and laparoscopic approaches had substantially more patients, given the more recent introduction of the robotic-assisted approach. This potential time-lead bias is inherent when comparing a new procedure with existing standards; however, it also offers the opportunity to examine the learning curve associated with the robotic platform, given that our study period includes the widespread and rapid dissemination of robotic-assisted ventral hernia repair that began around 2014 to 2015. Second, while results for ventral and umbilical hernia repair are similar, our results should be interpreted within the context that we are unable to account for hernia size. Third, our study focuses exclusively on Medicare patients. This demographic may not represent the broader population, and the results might not be generalizable to other non-Medicare patients. However, Medicare patients are likely to undergo ventral hernia repair both due to advanced age and likelihood of developing incisional hernias from prior surgery. We attempted to correct our surgeon volumes by proportionally inflating them based on the percentage of Medicare patients at each surgeon’s hospital. Although limited to Medicare data, this study has the unique strengths of being population-based and tracking longitudinal outcomes due to universal enrollment and low disenrollment. However, we could not distinguish fee-for-service patients using the American Hospital Association Annual Survey data, potentially leading to an overestimation of the total number of patients per surgeon when estimating the number of cases needed for surgeons’ learning curves.

A further limitation is that the data do not indicate whether the initial hernia repair during the study period was for a recurrent hernia. To address this, we excluded patients who had undergone hernia surgery in the 2 years before their first identified repair during the study period. Additionally, to ensure only the first index hernia repair for each patient was included, we excluded all subsequent hernia reoperations after identifying the index surgery. Another consideration is that our dataset only captures inpatient robotic ventral hernia repairs, excluding outpatient procedures, which are common for minimally invasive surgical procedures. The sample may not fully reflect the total volume of robotic-assisted hernia repairs. However, inpatient cases are often more complex or involve higher-risk patients, making the learning curve particularly relevant. Thus, our findings still offer valuable insights into surgeon performance in cases that require higher levels of expertise.

## Conclusions

In this national study covering the years 2010 to 2020 and reflecting a large group of surgeons and practice patterns, increasing experience with robotic-assisted ventral hernia repair was associated with lower long-term reoperation rates for hernia recurrence. Despite this, only a small proportion of surgeons overcame the learning curve to reach equivalence with open or laparoscopic approaches.

## Notes

Supplement 1. **eTable 1. ***Current Procedural Terminology* (*CPT*) Codes and *International Classification of Diseases Ninth and Tenth Edition* (*ICD-9* and *ICD-10*) Codes for Ventral Hernia **eTable 2.** Number of Patients by Year and Operative Approach
eMethods.
**eFigure.** Learning Curves Generated by the Linear Spline Approach for Robotic-Assisted Ventral Hernia Repair (4-Year Reoperation Rates for Hernia Recurrence, 2011-2017)

Supplement 2.
Data Sharing Statement
