# Supplementary material for: Learning Curves for Robotic-Assisted Ventral Hernia Repair
Source: JAMA Netw Open. 2024 Dec 3;7(12):e2448521. doi: 10.1001/jamanetworkopen.2024.48521 (PMC11615711; doi:10.1001/jamanetworkopen.2024.48521)
Supplement: Supplement 1. — eTable 1. Current Procedural Terminology (CPT) Codes and International Classification of Diseases Ninth and Tenth Edition (ICD-9 and ICD-10) Codes for Ventral Hernia eTable 2. Number of Patients by Year and Operative Approach eMethods. eFigure. Learning Curves Generated by the Linear Spline Approach for Robotic-Assisted Ventral Hernia Repair (4-Year Reoperation Rates for Hernia Recurrence, 2011-2017) [file jamanetwopen-e2448521-s001.pdf]

Supplemental Online Content

Loh WS, Howard RA, Fry BT, et al. Learning curves for robotic-assisted ventral hernia repair. *JAMA Netw Open*. 2024;7(12):e2448521. doi:10.1001/jamanetworkopen.2024.48521

eTable 1. *Current Procedural Terminology* (*CPT*) Codes and *International Classification of Diseases Ninth and Tenth Edition* (*ICD-9* and *ICD-10*) Codes for Ventral Hernia

eTable 2. Number of Patients by Year and Operative Approach

eMethods

eFigure. Learning Curves Generated by the Linear Spline Approach for Robotic-Assisted Ventral Hernia Repair (4-Year Reoperation Rates for Hernia Recurrence, 2011-2017)

This supplemental material has been provided by the authors to give readers additional information about their work.

© 2024 Loh WS et al. *JAMA Network Open*.

eTable 1. *Current Procedural Terminology* (*CPT*) Codes and *International Classification of Diseases Ninth and Tenth Edition* (*ICD-9* and *ICD-10*) Codes for Ventral Hernia

**ICD-9 Diagnosis Codes**	551.1, 551.2, 551.20, 551.21, 551.29, 552.1, 552.2, 552.20, 552.21, 552.29, 553.1, 553.2, 553.20, 553.21, 553.29
**ICD-9 Procedure Codes**	53.4, 53.41, 53.42, 53.43, 53.49, 53.5, 53.51, 53.59, 53.6, 53.61, 53.62, 53.63, 53.69
**ICD-10 Diagnosis Codes**	K42, K42.0, K42.1, K42.9, K43, K43.0, K43.1, K43.2, K43.6, K43.7, K43.9
**ICD-10 Procedure Codes**	0WQF0ZZ, 0WMF0ZZ, 0WQF0ZZ, 0WQF3ZZ 0WQF4ZZ, 0WQFXZZ, 0WUF07Z, 0WUF0JZ, 0WUF0KZ, 0WUF47Z, 0WUF4JZ, 0WUF4KZ
**CPT Codes**	49560, 49561, 49585, 49587, 49652, 49653, 49654, 49655, 49570, 49572

eTable 2. Number of Patients by Year and Operative Approach

**Year of surgery**	**All**	**Robotic**	**Laparoscopic**	**Open**
	**Number (% within year)**			
• 2010	20,074 (100.0)	414 (2.1)	4,773 (23.8)	14,887 (74.2)
• 2011	19,385 (100.0)	515 (2.7)	4,517 (23.3)	14,353 (74.0)
• 2012	18,072 (100.0)	574 (3.2)	4,240 (23.5)	13,258 (73.4)
• 2013	16,621 (100.0)	622 (3.7)	3,835 (23.1)	12,164 (73.2)
• 2014	15,080 (100.0)	785 (5.2)	3,262 (21.6)	11,033 (73.2)
• 2015	14,184 (100.0)	1,030 (7.3)	2,792 (19.7)	10,362 (73.1)
• 2016	13,645 (100.0)	1,417 (10.4)	2,533 (18.6)	9,695 (71.1)
• 2017	13,206 (100.0)	1,758 (13.3)	2,186 (16.6)	9,262 (70.1)
• 2018	11,585 (100.0)	1,821 (15.7)	1,774 (15.3)	7,990 (69.0)
• 2019	10,763 (100.0)	1,956 (18.2)	1,517 (14.1)	7,290 (67.7)
• 2020	7,915 (100.0)	1,730 (21.9)	942 (11.9)	5,243 (66.2)

© 2024 Loh WS et al. *JAMA Network Open*.

eMethods

We conducted a sensitivity analysis using 4-year reoperation rates for hernia recurrence, based on index surgeries from 2011 to 2017, to capture the full duration of the patient follow-up period. Surgeons needed to perform 15 robotic-assisted ventral hernia repairs to achieve outcomes equivalent to the national laparoscopic benchmark (eFigure). For the open approach, 12 robotic-assisted repairs were required to reach equivalent reoperation rates for hernia recurrence. Using this benchmark, only 7.4% of surgeons achieved lower patient reoperation rates than those with the laparoscopic approach, and 10.79% surpassed the rates of the open approach. However, this approach is limited as it may estimate artificially short learning curves due to smaller differences in reoperation rates for hernia recurrence with shorter follow-up periods.

eFigure. Learning Curves Generated by the Linear Spline Approach for Robotic-Assisted Ventral Hernia Repair (4-Year Reoperation Rates for Hernia Recurrence, 2011-2017)

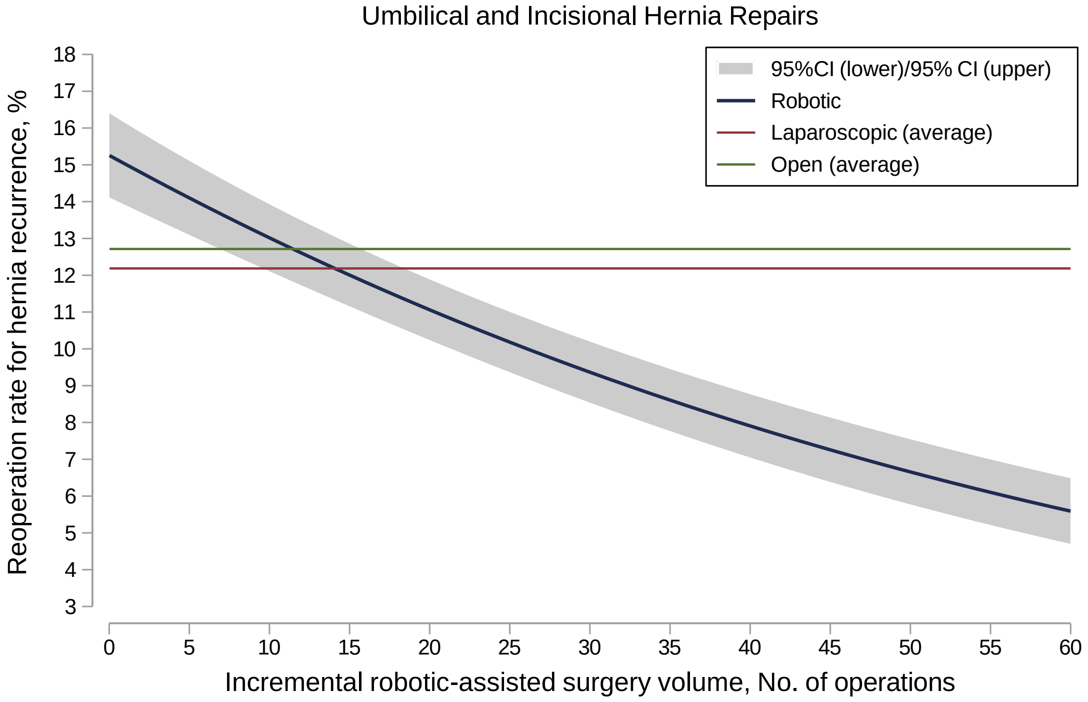

© 2024 Loh WS et al. *JAMA Network Open*.
