# Supplementary material for: Learning Curves for Robotic-Assisted Ventral Hernia Repair
Source: JAMA Netw Open. 2024 Dec 3;7(12):e2448521. doi: 10.1001/jamanetworkopen.2024.48521 (PMC11615711; doi:10.1001/jamanetworkopen.2024.48521)
Supplement: Supplement 2. — Data Sharing Statement [file jamanetwopen-e2448521-s002.pdf]

Data Sharing Statement

Loh. Learning Curves for Robotic-Assisted Ventral Hernia Repair. *JAMA Netw Open*.
Published December 03, 2024. doi:10.1001/jamanetworkopen.2024.48521

Data
Data available: No
